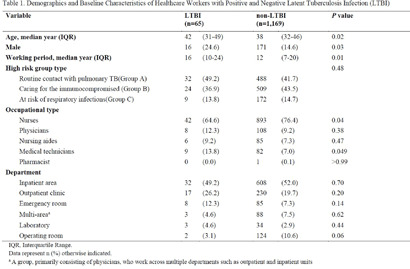# Latent Tuberculosis Conversion Rates and Characteristics of Converters Among Healthcare Workers in High-Risk Departments

**DOI:** 10.1017/ash.2025.365

**Published:** 2025-09-24

**Authors:** Seung Hee Ryu, Sun-Kyung Kim, Jeeyoon Kim, Jiwon Jung, Kyung-Wook Jo, Tae Sun Shim, Yong Pil Chong

**Affiliations:** 1 Asan medical center

## Abstract

**Background:** The Republic of Korea ranks second among OECD countries for tuberculosis (TB) incidence. National TB control guidelines mandate latent TB infection (LTBI) screening and treatment for healthcare workers (HCWs), especially those in high-risk departments. At our 2,700-bed tertiary hospital in Seoul, annual LTBI screening and treatment have been actively implemented since 2017, targeting HCWs at elevated risk of TB exposure. This study evaluates LTBI conversion rates among high-risk HCWs and characteristics of HCWs with conversion (converters) over the past five years. **Methods:** Following national guidelines, HCWs were classified into three high-risk groups: those likely to have routine contact with pulmonary TB patients (Group A), those caring for immunocompromised patients (Group B), and those at risk of respiratory infections despite no routine TB contact (Group C). Annual screening included interferon-gamma release assay (IGRA) and chest radiography. HCWs with positive IGRA results (≥0.35 IU/m) were strongly encouraged to undergo latent tuberculosis treatment. We analyzed data from HCWs working in high-risk tuberculosis units who had worked for more than five years from 2020 to 2024. HCWs with prior IGRA positivity were excluded. **Results:** Among, 1467 HCWs, 15.9% (233/1,467) had been diagnosed with LTBI before 2020, while the cumulative LTBI conversion rate between 2020 and 2024 was 5.3% (65/1,234). The annual LTBI conversion rates ranged between 0.7% and 1.5%. The median age of converters was 42 years, significantly older than non-converters (median 38 years; P = 0.02). Male converters comprised 24.6% (16/65) compared to 14.6% (171/1,169) in the non-converter group (P = 0.03). Longer tenure was observed among converters (median 16 years) than non-converters (median 12 years; P = 0.01). Although medical technicians and emergency room staff exhibited higher conversion rates, these differences were not statistically significant. Among LTBI cases, 78.8% completed treatment, with 9.1% demonstrating reversion. The annual incidence of active tuberculosis among HCWs at our hospital significantly declined to an average of 0.2 cases per year between 2020 and 2024, compared to 4.4 cases per year between 2015 and 2019 **Conclusions:** Annual LTBI screenings revealed conversion rates of approximately 1%, primarily affecting older, long-tenured, and male HCWs. Active LTBI treatment effectively reduced the risk of active TB among hospital staff.

**Figure:**